# Enhanced osteogenic differentiation of stem cells by 3D printed PCL scaffolds coated with collagen and hydroxyapatite

**DOI:** 10.1038/s41598-022-15602-y

**Published:** 2022-07-20

**Authors:** Zahra Ebrahimi, Shiva Irani, Abdolreza Ardeshirylajimi, Ehsan Seyedjafari

**Affiliations:** 1 Department of Biology, Science and Research Branch, Islamic Azad University, Tehran, Iran; 2 Urogenital Stem Cell Research Center, Shahid Beheshti University of Medical Sciences, Tehran, Iran; 3 Department of Biotechnology, College of Science, University of Tehran, Tehran, Iran

**Keywords:** Stem cells, Biomedical engineering

## Abstract

Bone tissue engineering uses various methods and materials to find suitable scaffolds that regenerate lost bone due to disease or injury. Poly(ε-caprolactone) (PCL) can be used in 3D printing for producing biodegradable scaffolds by fused deposition modeling (FDM). However, the hydrophobic surfaces of PCL and its non-osteogenic nature reduces adhesion and cell bioactivity at the time of implantation. This work aims to enhance bone formation, osteogenic differentiation, and in vitro biocompatibility via PCL scaffolds modification with Hydroxyapatite (HA) and Collagen type I (COL). This study evaluated the osteosupportive capacity, biological behavior, and physicochemical properties of 3D-printed PCL, PCL/HA, PCL/COL, and PCL/HA/COL scaffolds. Biocompatibility and cells proliferation were investigated by seeding human adipose tissue-derived mesenchymal stem cells (hADSCs) onto the scaffolds, which were analyzed by 3-(4,5-dimethylthiazol-2-yl)-2,5-diphenyl tetrazolium bromide (MTT) assay, and 6-diamidino-2-phenylindole (DAPI) staining. In addition, the bone differentiation potential of the hADSCs was assessed using calcium deposition, alkaline phosphatase (ALP) activity, and bone-related protein and genes. Although all constructed scaffolds support hADSCs proliferation and differentiation, the results showed that scaffold coating with HA and COL can boost these capacities in a synergistic manner. According to the findings, the tricomponent 3D-printed scaffold can be considered as a promising choice for bone tissue regeneration and rebuilding.

## Introduction

Bone injuries are caused by infections, accidents, pathological destruction, trauma, and congenital disabilities^1,2^. In order to fully replace damaged bone, artificial bone grafts should have a structure that can adapt to the specific patient's needs. Adaptable artificial bone grafts can replace damaged bone. The applications of tissue engineering have become increasingly widespread in the medical field^3^. The main aim of tissue engineering is to repair and restore injured tissues via the use of cells, growth factor, and scaffolds made from biodegradable and biocompatible synthetic or natural materials^4,5^. Among them, the three-dimensional (3D) scaffold has significantly impacts on mass transport, proliferation, and differentiation of cells. In addition, the inner scaffold structure plays an important role in bone regeneration. Also, scaffolds' mechanical strength and physical properties can mimic the extracellular matrix (ECM)better and present a suitable porosity for cell attachment and growth^6–8^. Therefore, the biological scaffold should be biodegradable, biocompatible, and non-toxic^9^. Several methods and technologies have been used to prepare porous scaffolds, including fused deposition modeling (FDM), selective laser sintering (SLS), stereolithography (SLA), inkjet printing (IP), laser metal deposition (LMD), and direct ink writing (DIW)^9,10^. One of the characteristics of 3D printing via fused deposition modeling (FDM) is controlling the porosity and pore size and melting the filament or polymer through the nozzle layer-by-layer on the structural plate, which leads to creating a 3D shape^11–13^.

Additive manufacturing (AM) or 3D printing techniques can be used to 3D print a wide range of synthetic and natural materials, such as metals, polymers, polymer composites, ceramics, and cement^14^. Poly(ε-caprolactone) (PCL) is a suitable synthetic polymer for use in bone tissue engineering because of its mechanical strength, thermoelastic behavior, adjustment of structural degradation time in proportion to recovery biocompatibility, and rapid freezing due to its calcium ions. However, PCL shows low performance, low flexibility, and lack of cell attachment sites, and there are no functional groups in the polymer chain compared with the natural bone tissue^9,15–17^. PCL easily combines with other synthetic and natural polymers and bioceramic materials to allow PCL properties to be precisely adjusted^18^. In contrast, hydroxyapatite (HA) is the most stable form of calcium phosphate at the body’s pH. Moreover, it is very suitable for bone substitute due to its excellent osteoconductivity, biocompatibility, cell binding, and proliferation^15–17^. Numerous experiments have been conducted using PCL and HA materials in combined biomedical scaffolds for bone tissue engineering applications^19,20^. To date, 3D printing PCL/HA bone scaffolds have been reported by Jiao et al.^19^ and Kim et al.^21^ by using FDM 3D printing. Both researchers observed an increase in the mechanical strength of composite scaffolds compared to PCL scaffolds alone. Therefore, PCL and HA are combined to provide the scaffold with the appropriate mechanical strength. The proper scaffold should have appropriate cell attachment, proliferation, bioactive properties, and sufficient mechanical strength  for primary support. By coating synthetic polymer scaffolds (such as poly(ε-caprolactone)) with some of the ECM components found in bone tissues, such as collagen, the poor cell attachment could be improved^22,23^. Collagen is a biocompatible protein commonly used in tissue engineering to improve the repair of damaged tissue due to its high capacity to strengthen cell attachment, proliferation, and differentiation^24–27^. Integrin and other molecules within the ECM, such as fibronectin, provide unique binding motifs for collagen to attach to the cells^22^. Li et al*.*^25^ reported that nHA/CoL scaffolds expressed more SOX9, OCN, and COL1A1 than COL scaffolds ( p < 0.05). Therefore, bone tissue engineering scaffolds require bioactive materials to mimic a similar structure of bone ECM^24–26^. Different properties of scaffolds help to stimulate cell differentiation into specific cell lines. A scaffold must have the proper chemical, physical, and osteoinductive properties to promote bone regeneration^28,29^. The selection of stem cells is an essential factor in the success of an ideal hybrid tissue-engineering scaffold. Mesenchymal stem cells (MSCs) are good candidates for osteochondral tissue engineering due to their differentiation capacity into different classes, such as osteoblasts and chondroblasts. It is essential to control the differentiation capacity of the MSCs without any ethical concerns^30^. The aim of this study was to  investigate the biological behavior of the human adipose derived stem cells (hADSCs) while cultured on a fabricated 3D PCL empty scaffold and while coated with HA nanoparticles and collagen type I (COL). The PCL scaffold as a backbone with high mechanical properties was printed using an FDM-type 3D printer. After plasma treatment, HA and COL were added to the scaffold to increase the scaffold’s osteointegration, mimic the bone microenvironment and improve its biocompatibility. Finally, the fabricated scaffolds were structurally characterized using FeSEM, XRD, ATR-FTIR, and compression tests.  Futthermore, the scaffolds osteo-supportive capacity was assessed by using common osteogenic genes and protein markers.

## Results

### Fabrication of scaffolds

**Figure 1:**
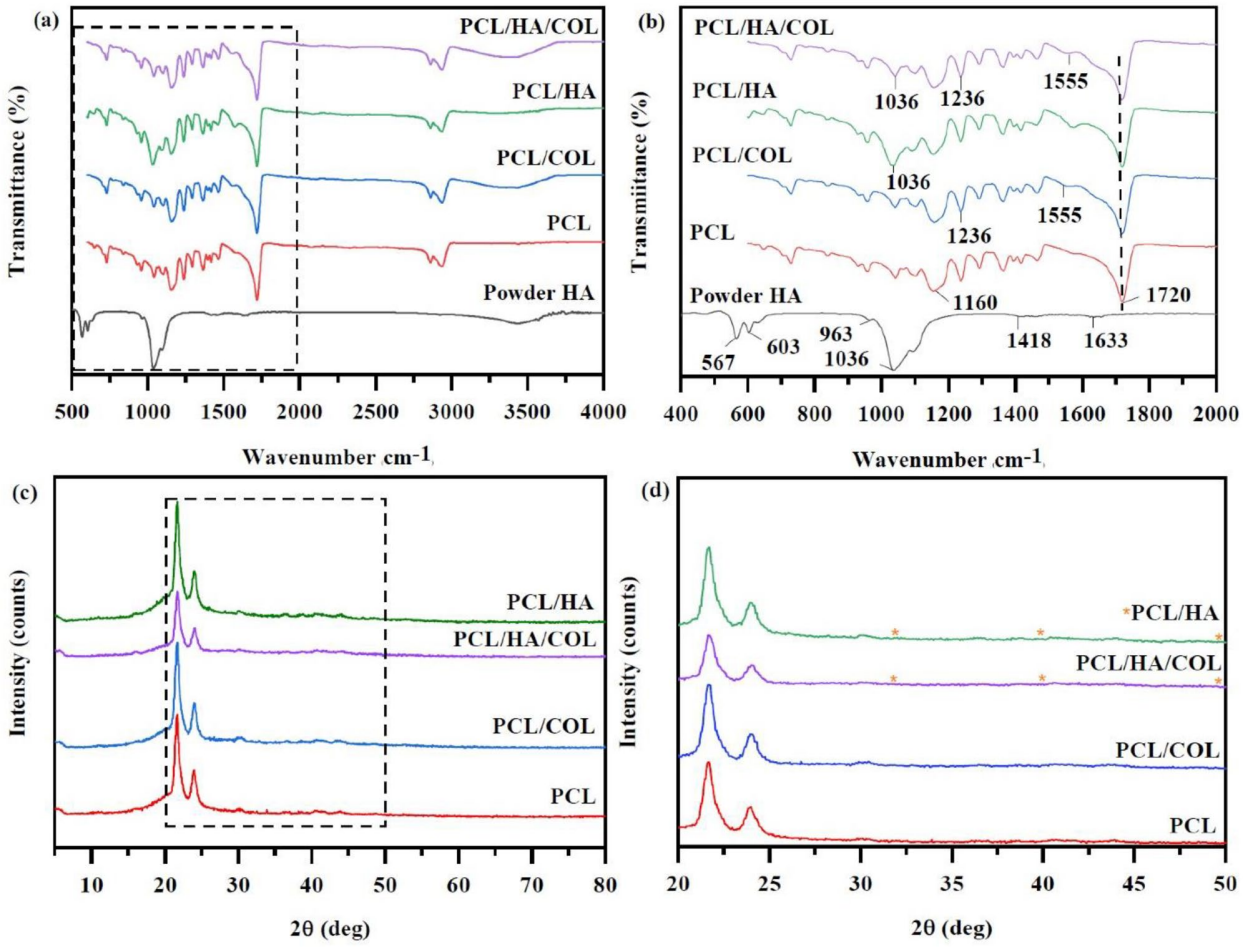
(**a**,**b**) ATR-FTIR spectra of powder HA, PCL scaffold, COL and HA-coated PCL scaffolds, and (**c**,**d**) The X-ray diffraction (XRD) spectra of PCL and composite scaffolds.

The FTIR spectrum was performed to evaluate the functional groups of different fabricated scaffolds. The spectra of FTIR composites scaffolds (PCL/HA, PCL/COL, PCL/HA/COL) and functional groups of raw materials (PCL, HA powder) are shown in Fig. 1. In the spectrum obtained from the PCL/HA/COL scaffold, the characteristic peaks of all materials can be seen in the composite scaffold. PO_4_^3−^ peaks of HA are in the range of 961–1090 cm^−1^ , with the maximum at 1037 cm^−1^^31^. As shown in Fig. 1a,b, pure PCL shows the main characteristics C = O of the carbonyl ester group at 1720 cm^−1^, which is consistent with the peaks observed in PCL/HA, PCL/COL, and PCL/HA/COL scaffolds. The carbonyl ester group (C = O) peaks of PCL were at 1720 cm^−1^, and CH2 asymmetric and symmetric tensions were at 2936 cm^−1^ and 2861 cm^−1^, respectively, and C-O bands were determined at around 1160 cm^−1^^20,32–34^. Regarding COL, the FTIR spectra (Fig. 1a,b) for the PCL/COL scaffold were determined to be NH coupled with hydrogen bond amide I strip with tensile vibration at about 3435 cm^−1^, and the tensile vibration of amide II (N–H) band and CN were observed at 1555 cm^−1^ and 1236 cm^−1^ respectively^35^. In general, an amid I strip is a sensitive indicator of the secondary structure^36^. The infrared spectrum of PCL/COL showed that COL was successfully coated on the surface of the PCL scaffold^26^.

XRD analysis was performed to evaluate the placement of the composition mineral particles on the surfaces of composite scaffolds, while raw PCL granules and HA powder were used as comparisons. XRD patterns of different scaffolds are presented in Fig. 1c,d. The typical peaks for the pure PCL scaffold appeared at (110), (200), and (111) that all found at around 2θ = 21.7°, 24.0°, and 22.3°, respectively. The characteristic peak was related to the structure of HA ; a reflection of (002), (210), (211), and (310) was at 2θ angles of 24.4, 27.25, 31.8, and 45.7, respectively^37^. In PCL/HA and PCL/HA/COL composite scaffolds, detectable HA diffraction peaks at were detected 31.9°, 39.9°, and 49.8°^38^.

**Figure 2:**
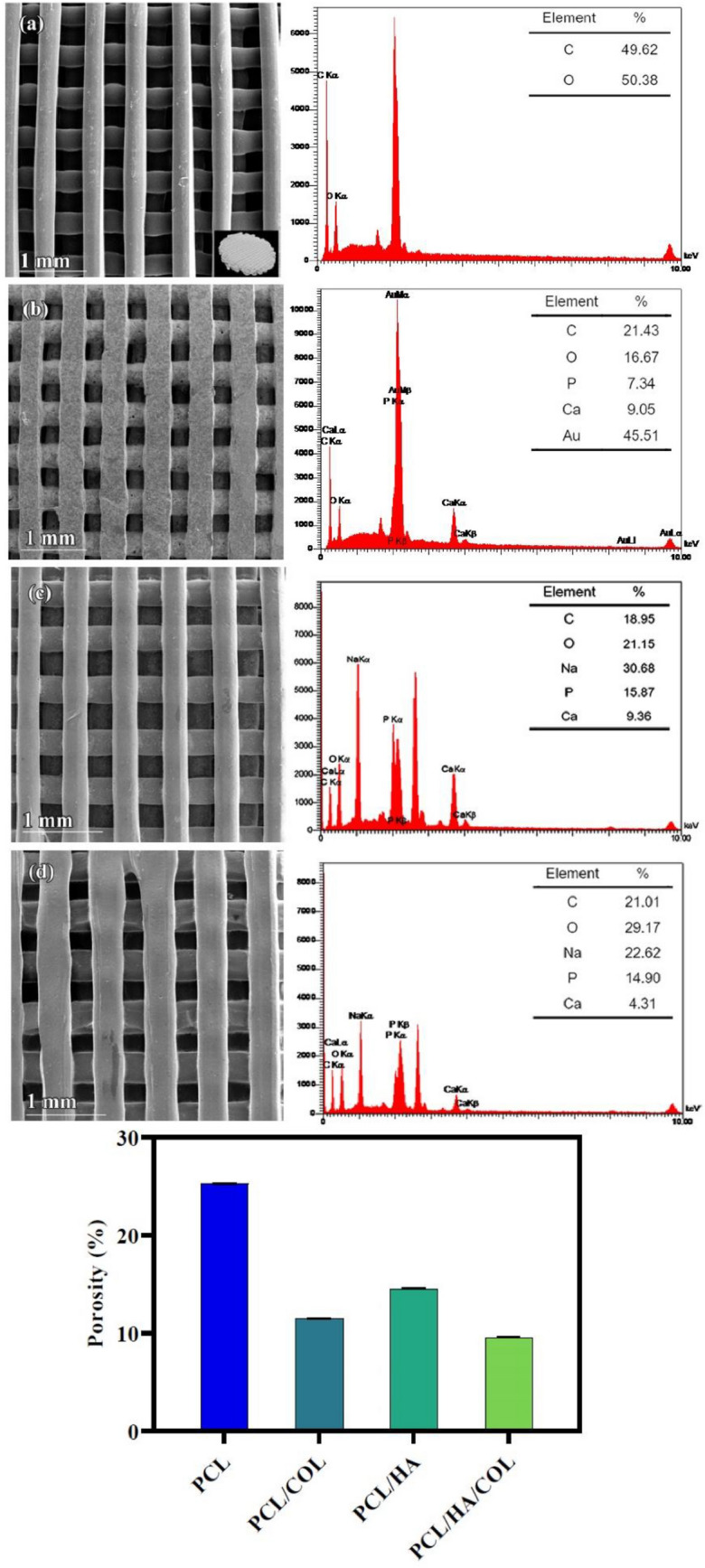
SEM images and EDS spectra analysis of the morphology (**a**) PCL, (**b**) PCL/HA, (**c**) PCL/COL, and (**d**) PCL/HA/COL scaffold and the change of porosity of the 3D printed scaffolds.

FeSEM observed the surface morphology and quality of the printed scaffolds, and the SEM images showed that the surfaces of the PCL scaffolds were smooth (Fig. 2a). The surface of PCL/HA and PCL/HA/COL scaffolds contained numerous white convex protrusions resulting from the inclusion of HA particles (Fig. 2b,d). The surface of PCL/HA and PCL/HA/COL scaffolds looked rougher than for PCL/COL and PCL scaffold surfaces, which were covered with many HA particles. SEM analysis revealed that the surface morphology of the PCL after COL coating was slightly wrinkled (Fig. 2c).

EDX was used to identify the elemental composition percentage on the surface of scaffolds. EDX confirmed the presence of calcium, carbon, phosphorus, and oxygen in the samples, which confirmed the coating of the HA and COL layers on the surface of PCL scaffolds (Fig. 2).

Due to the gold plating, the presence of Au was observed in the samples. The diameter of all scaffold filaments was between 324–369 μm, also determined by SEM images (Table 1).

**Table 1:** The properties of the scaffolds.

Scaffold	Average diameter (µm)	Pore size (µm)	Porosity
PCL	324 ± 14	317 ± 22	25.27
PCL/COL	299 ± 25	331 ± 18	11.52
PCL/HA	350 ± 24	262 ± 12	14.57
PCL/HA/COL	369 ± 10	287 ± 7	9.61

**Figure 3:**
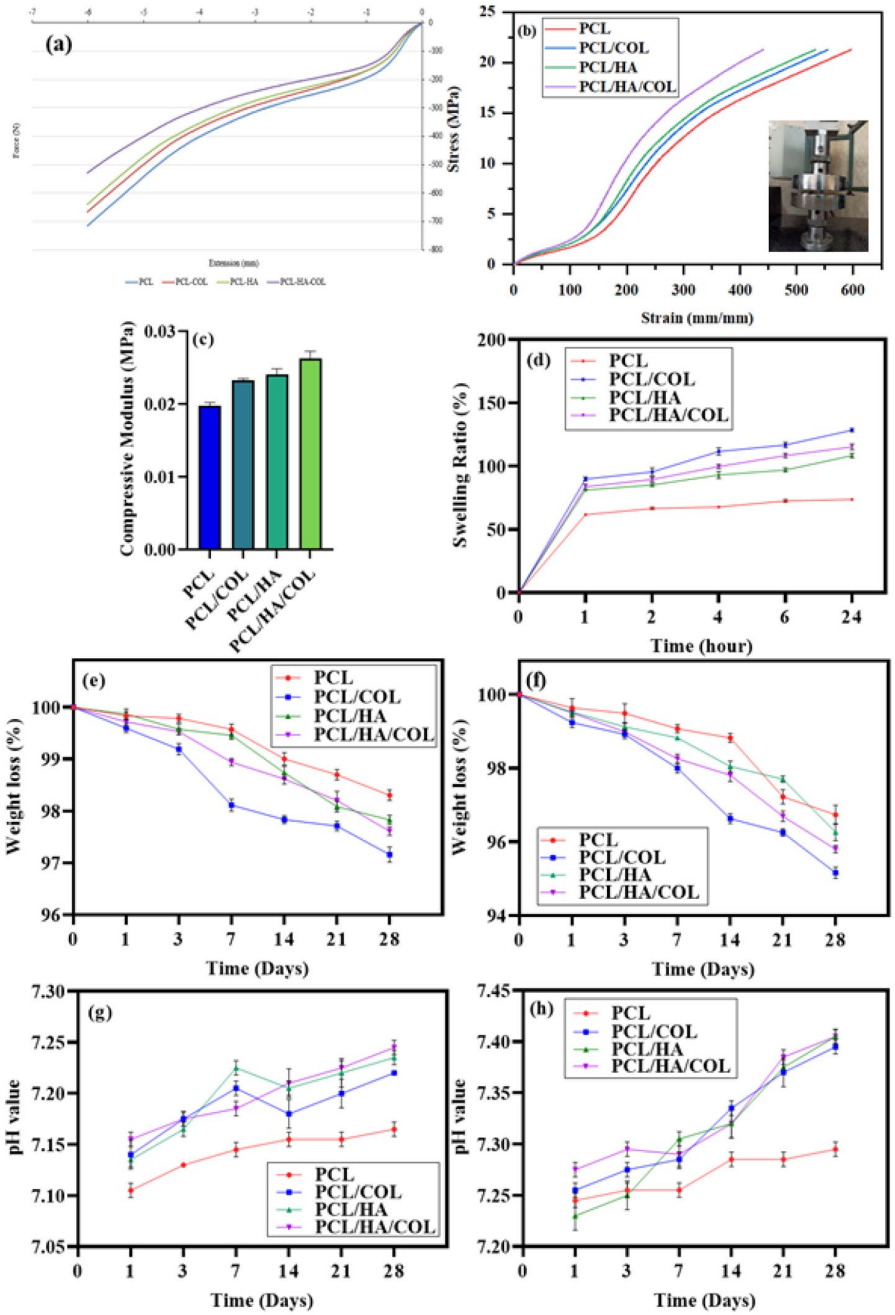
(**a**) Force-extension diagram, (**b**) Stress–strain diagram, and (**c**) compressive modulus for the different scaffolds. (**d**) Variation of swelling behavior of the flat-printed FDM scaffolds. (**e**) Weight loss of PCL, PCL/HA, PCL/COL and PCL/HA/COL scaffold during soaking in PBS and (**f**) PBS-Lipase for 28 days. (**g**) pH values of the scaffolds during the degradation in PBS and (**h**) lipase. Measurements were started after 1 day, day 0 control = pH 7,4. (n = 3 in each group, *p ≤ 0.05).

The results of the scaffold compression test are shown in Fig. 3a–c where their mechanical properties differed among the various fabricated scaffolds. The Young’s modulus of the PCL, PCL/HA, PCL/COL, and PCL/HA/COL scaffolds were determined at 0.0175 MPa, 0.0213 MPa, 0.0216 MPa, and 0.0258 MPa, respectively. However, PCL scaffolds were characterized by the lowest values for Young's modulus (0.0175 MPa).

The tissue engineering scaffolds must absorb water well so thoroughly wet^17^. The PCL scaffold's hydrophilicity is relatively low^39^. The effect of COL and inorganic components (HA) on the water adsorption of the scaffolds was investigated. Pure PCL scaffolds absorbtion less water than PCL/COL, PCL/HA, and PCL/HA/COL composite scaffolds measured swelling test (p ≤ 0.01) (Fig. 3d).

We studied the behavior of the degradation of all printed scaffolds while incubated in a lipase solution and phosphate buffer to simulate enzymatic degradation Fig. 3e,f shows the mass loss of scaffolds tested over 28 days. A pure PCL scaffold was also evaluated as a control to measure the effect of HA and COL on the degradation mechanism. Compared with the initial scaffold weight, the scaffolds lost weight within 7 days, and then the destruction of the scaffold was gradual until the end of 28 days. Unlike composite scaffolds, the pure PCL scaffolds showed less degradation. The results showed the influential role of COL on the rate of scaffold degradation. We investigated the acidification of the media after immersion in PBS and PBS-Lipase solution for 28 days during the degradation experiments (Fig. 3g,h).

### Biocompatibility of scaffolds

**Figure 4:**
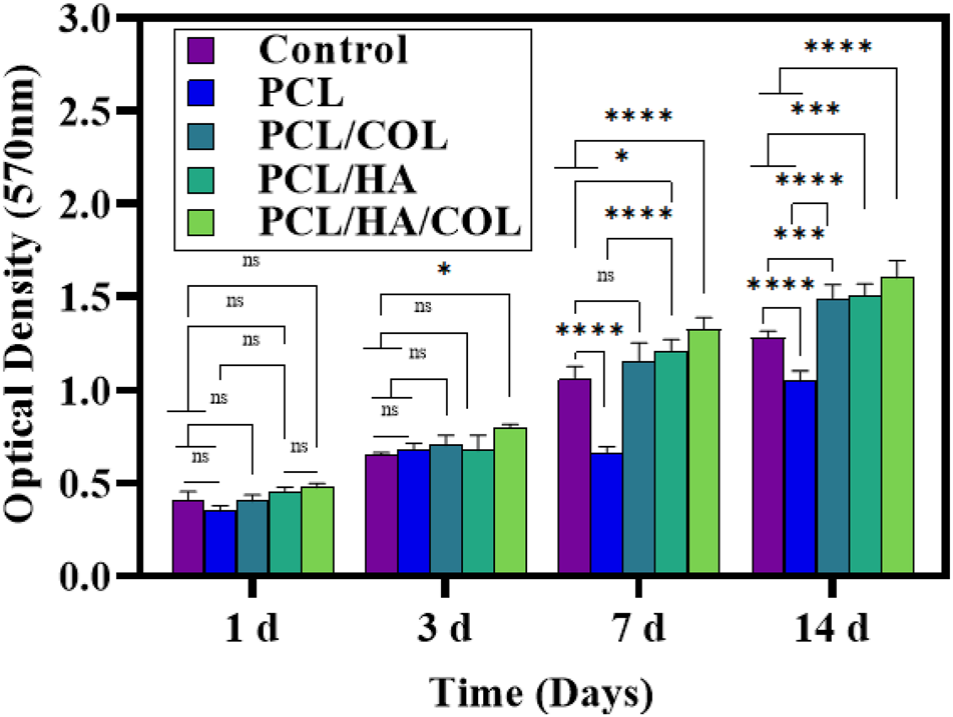
The proliferation rate of hADSCs on the scaffolds after 1,3,7 to 14 days, and determined by MTT assay. (n = 3, *P ≤ 0.05, **P ≤ 0.01, ***P ≤ 0.001, ****P ≤ 0.0001).

Biocompatibility of the fabricated scaffolds compared with control was evaluated via MTT assay (Fig. 4). hADSCs cells were cultured on all four scaffolds (culture times: 1, 3, 7, and 14 days). The results showed an increasing trend in the proliferation rate of the cells cultured on the scaffold from day 3 to day 14. Compared with the PCL scaffold, cell proliferation on the composite scaffold was significantly different. In addition, when comparing day 1 and day 3, there was no statistically significant difference in cell proliferation between groups. A similar proliferation pattern was detected until the third day. The proliferation rate of the cells increases with HA and COL coating on the PCL scaffolds during the longer incubation time. The cells cultured on the PCL/HA, PCL/COL, and PCL/HA/COL scaffolds showed a more positive (p ≤ 0.0001) effects on cell proliferation than those cultured on the pure PCL. Therefore, 3D printed scaffolds did not exhibit cytotoxicity during stem cell proliferation. Cumulatively, composites scaffolds are compatible scaffolds for bone tissue engineering.

### Osteoinductivity of scaffolds

**Figure 5:**
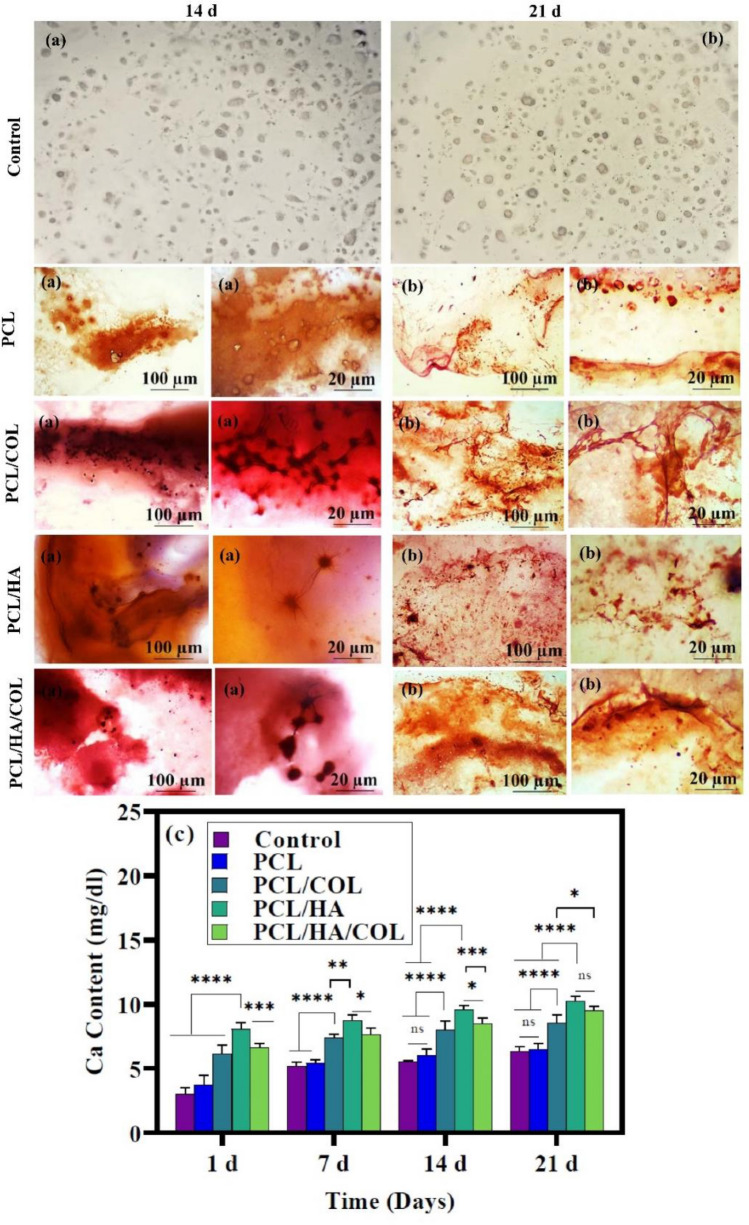
Bone mineralization capacity. In the following image, (**a**) Alizarin Red staining after a 14 days' culture. (**b**) After 21 days, Alizarin Red staining. After 21 days of culture, more staining was observed (Scale bar = 100 μm and 20 μm). (**c**) Calcium concentration of hADSCs on the scaffolds measured after 1,714 and 21 days' culture (n = 3, ***p ≤ 0.001).

Alizarin red staining was performed to qualitatively evaluates the deposited calcium on the different fabricated scaffolds (Fig. 5). The amount of mineralization in control cultures was lower than in the other groups. However, the intensity of red spots in the PCL/HA and PCL/HA/COL scaffolds during 21 days is higher than in other groups, which indicated the development of mineralization in the simultaneous presence of HA and COL. The quantity of calcium generated by differentiated hADSCs in various scaffolds was measured via calcium content assay on days 1,7,14, and 21. The calcium content of the PCL/HA scaffold was significantly higher than for other scaffolds on all days (Fig. 5c). After PCL/HA scaffold, the highest calcium content was related to the PCL/HA/COL composite scaffolds. Although it was not significantly different with PCL/HA scaffolds, a significant difference was observed between the calcium content of PCL/HA/COL scaffolds and pure PCL, PCL/COL, and control groups.

**Figure 6:**
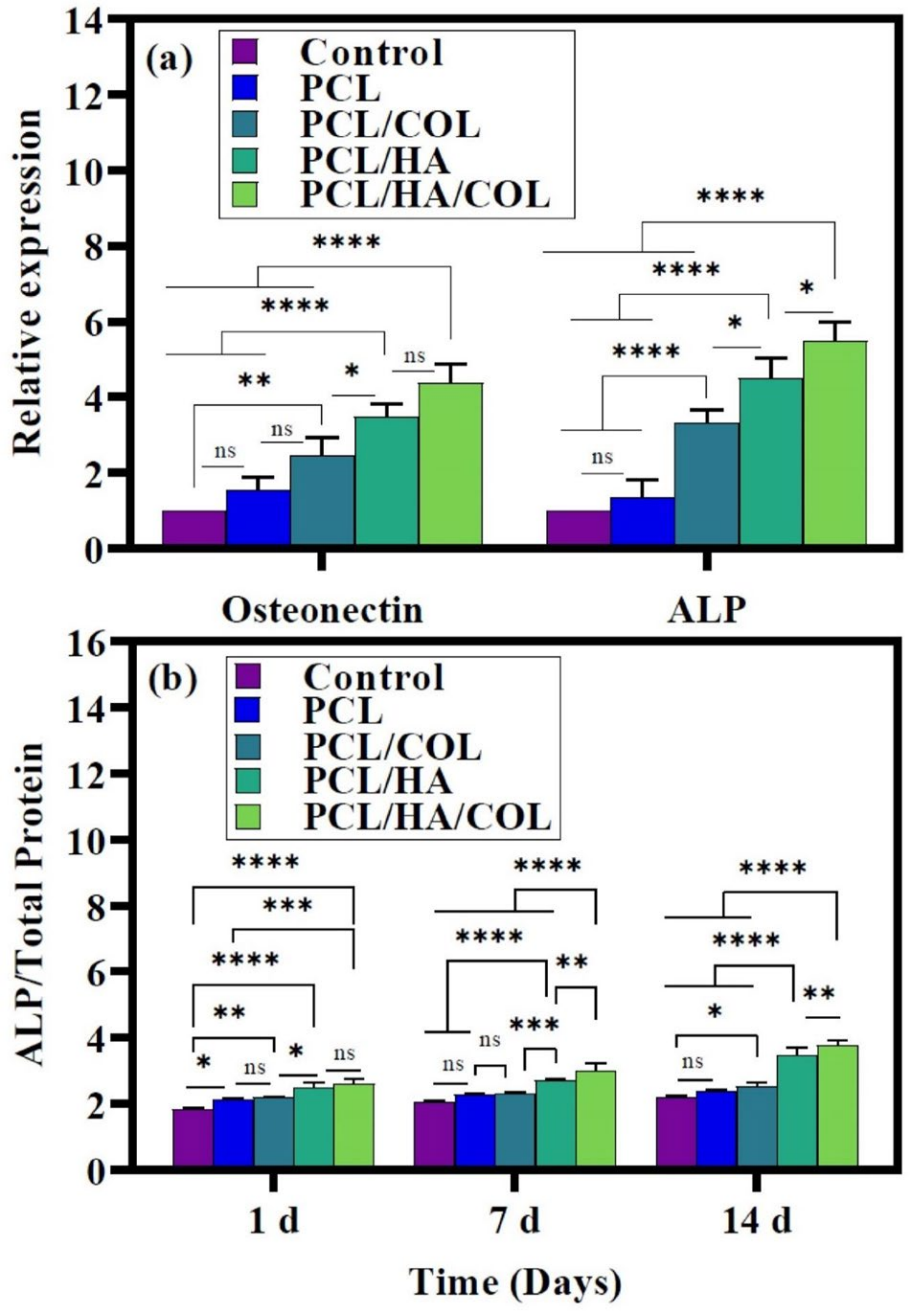
(**a**) mRNA Expression of osteogenic related genes on scaffolds, Osteonectin (first column) and ALP (second column) after 21 days (n = 3, *p ≤ 0.05 vs. Control group). (**b**) ALP activity of hADSCs cells seeded on scaffolds in osteogenic medium.

To assess the osteogenic differentiation characteristics of hADSCs seeded on the scaffolds, real-time PCR was used to determine the expression levels of ALP and Osteonectin mRNA (Fig. 6a). Expression levels of these genes were higher in the hADSCs cultured on the different scaffolds than in the control group. The results indicated that the highest level of these genes was detected in the hADSCs cultured on the PCL/HA/COL scaffolds compared to others. The results demonstrated that with HA/COL immobilization on the PCL scaffolds surface, bone differentiation of the hADSCs increased significantly. Similar observations have been reported for improving the surface condition of polymer scaffolds^25,33,39,40^. A higher bone markers expression was found in cultured cells on PCL/HA/COL and PCL/HA scaffolds.

The ALP activity of ADSC cells cultured on the pure PCL scaffolds and composite scaffolds under an osteogenic differentiation medium was evaluated on days 1, 7, and 14 (Fig. 6b). The results showed a significant increase (p < 0.0001) in ALP activity from days 1 to 14 for the PCL/HA/COL composite scaffold, which could explain the scaffold's ability to enhance bone differentiation. Composite scaffolds showed the highest ALP activity on day 14 due to the synergistic effect of HA and COL.

**Figure 7:**
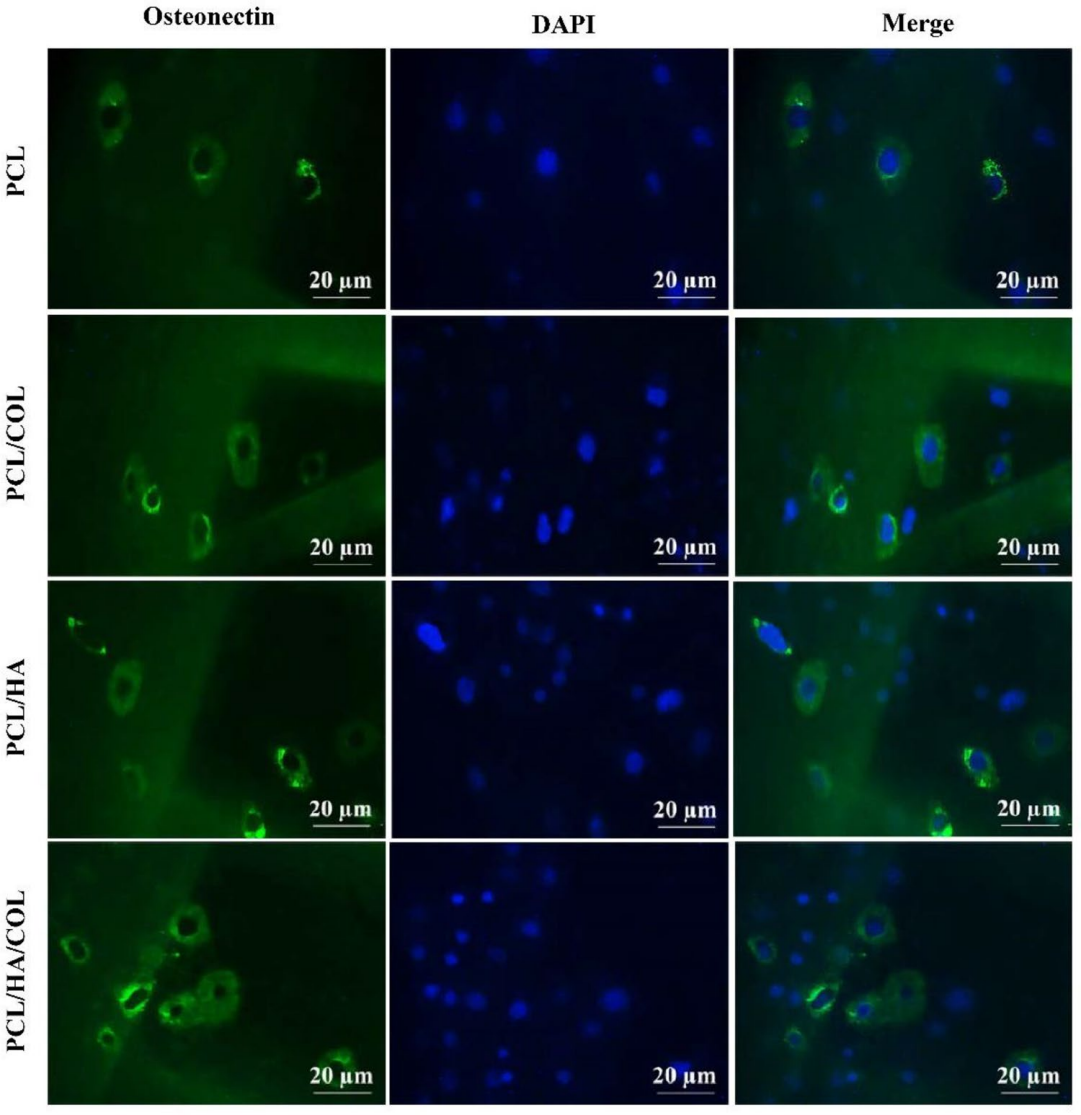
The expression pattern of osteogenic markers Osteonectin (first column), for hADSCs on PCL, PCL/HA, PCL/COL and PCL/HA/COL scaffold after 21 days’ incubation in osteogenic medium determined by ICC assay. Cell nuclei in the images are stained with 4′,6- diamidino-2-phenylindole (DAPI) (Scale bar represents 20 μm).

ICC test was performed to confirm Osteonectin expression as a bone-specific protein marker at the translation level in the hADSCs cultured on the scaffolds after 21 days of incubation (Fig. 7). ICC image analysis showed that Osteonectin expression did not occur for cells seeded on the pure PCL scaffold while it was detected in the composite scaffolds in the presence of HA and COL. Higher expression level of Osteonectin in the cells cultured on the PCL/HA/COL scaffold showed that a combination of HA and COL along with the 3D structure of the PCL scaffold could be more successful. In addition, it can be noted that the composite scaffolds can support protein expression for up to 21 days.

## Discussion

Tissue engineering appears to be a promising approach to overcoming the challenges of traditional bone graft treatments. Scaffolds for tissue engineering have been widely studied^41^. As materials design and polymer chemistry advance, 3D printing could revolutionize healthcare significantly beyond personalized implants^42^. In solid-freeform fabrication, FDM is the most widely used due to (i) its cost-effectiveness, (ii) uses a variety of materials, and (iii) its ability to patient-specific defects^7,43^. 3D printing technologies can be used in a variety of polymers like polylactic acid (PLA), poly(ε-caprolactone) (PCL), and poly(lactic-co-glycolic) acid (PLGA)^44^. One of the biocompatible polymers is poly(ε-caprolactone) (PCL), widely used in the tissue engineering field for its mechanical strength, good biocompatibility, degradability, and suitability for modification. PCL's insufficient bioactivity makes it unsuitable for bone tissue engineering^19^. So, to improve cell-PCL interactions, several surface modifications such as alkaline hydrolysis with sodium hydroxide (NaOH), plasma treatments, and coating the surface of 3D printed PCL scaffolds have been introduced^45^. Surface modification with different biomaterials based on calcium, silicate, and phosphate such as hydroxyapatite (HA), calcium silicate (CS), bioactive glass (BG), and tri-calcium phosphate (TCP) have been widely evaluated. Direct bonds between the biomaterials and the living bones are possible after implant^46^. In addition, surface coating is a modification method used to increase the mechanical strength, bioactivity, hydrophilicity, and cell compatibility of scaffolds^47^. According to Park et al., in 2021, surface modification of 3D-printed PCL/HA scaffolds using O2 plasma and sodium hydroxide increased hydrophilicity and PCL/HA scaffold protein adsorption capacity, which support cell attachment, proliferation, and osteogenesis of human dental pulp derived stem cells (hDPSCs)^48^. This study aimed to construct a 3D printing scaffold suitable for bone repair and regeneration. We successfully constructed PCL scaffolds as the primary material with controllable structure and porous morphology via 3D printing. We also examined the effect of surface treatment of 3D-printed PCL scaffolds with HA and COL on the hADMSCs differentiation and osteogenesis.

This exploratory study shows that the materials used in the fabrication of tissue scaffolds affect the properties of the fabricated scaffolds. The decrease in the strength of PCL peaks in the PCL/HA/COL scaffold is related to the amorphous COL and HA coating on the surface of the PCL scaffolds. For a comparative analysis, SEM images of scaffolds after immersion in HA and COL are presented along with EDX data. The coated scaffolds displayed a rough condensation surface attributed to the HA and COL layers. The addition of HA and COL changed the surface morphology of the PCL scaffolds. HA and COL are components of natural bone tissue that stimulate cell proliferation on the scaffold, and scaffold SEM results showed additional surface area for cell attachment^49,50^. Therefore, the HA/COL coating can provide a bone-like surface for PCL scaffolds and accelerate healing. A study by Deligianni et al. demonstrated that the inclusion of hydroxyapatite increases the surface roughness of the PCL scaffold. Their result revealed that the surface roughness increases cell binding and proliferation^5^.

One of the main benefits of 3D printing is the ability to print scaffolds with porous and complex structures, so large pores and high porosity encourage the organization of new bone within the human body and promote implant osseointegration. The porous network of scaffolds with interconnected pore structures helps cell growth, promotion, and migration^51^. The minimum suggested pore measure for the scaffold is between 100 and 150 µm. However, some studies have shown better osteogenesis, cell proliferation, migration, differentiation, and nutrient transfer when implants have pores greater than 300 µm^19,27,52^. Karageorgiou and Kaplan demonstrated that the minimum pore measure should not be smaller than 100 µm. Additionally, larger pores promote the growth of bone by increasing vascularity^53^. Nevertheless, increasing the scaffold's pore size reduces the structure's mechanical completeness^19,52^. The PCL scaffold's pore size and filament diameters were reduced while composited with COL and HA. However, the addition of COL on the scaffold did not significantly reduce the porosity of the PCL scaffold. In addition, the average pore size is also consistent with the porosity reduction process. The pores in the micrographs are interconnected. The measurements were performed with Image J software on SEM images to obtain pore size and filament diameters (Table 1).

Linear behavior related to the elastic response was observed in the first stage. The collapse of the pores under pressure and the compaction of the scaffold under almost constant stress were observed in the second and third stages. The slope of the linear part of the stress–strain curve was defined as the compressive modulus^40^. The PCL scaffold has a rubbery state, and increasing the HA content results in a rise in the scaffold's mechanical properties, while high amounts of HA lead to reduced porosity and pore size^54^. HA-containing composite scaffolds had a higher modulus than pure PCL scaffolds, indicating that the inherent strength of HA can help to enhance the composite scaffold's mechanical properties. Compressive moduli of the PCL/HA/COL significantly improved compared to PCL/COL and pure PCL with Young's modulus of 0.0258 MPa. It has been found that material and process parameters significantly affect FDM's mechanical properties^55^.

A wet surface with suitable chemical properties can support cell adhesion, proliferation, differentiation, and migration^36^. Consequently, it appears that incorporating hydrophilic inorganic materials (HA) into PCL matrices may prove an effective method for increasing the hydrophilicity of the polymer. The high level of hydroxyl groups in HA may enable the substance to absorb water. Therefore, HA can be increased to improve the hydrophilicity of the composites.

The hydrophobicity and water absorption can also affect the destruction of membranes in the biological environment. The higher weight loss of the COL containing membranes compared with pure PCL scaffolds could be due to the more excellent hydrophilicity of the scaffolds and the hydrolysis of the COL carboxylamine groups. Polymer composites degrade through three stages: (1) hydrating the polymer, (2) cleaving ester bonds, and (3) diffusing soluble components^56^. The target of the lipase enzyme is the oxygen and carbonyl groups in the lipids. Brugmans et al. examined the enzymatic digestion of PCL scaffolds derived through an electrospinning procedure. They found that when scaffolds were treated with lipase for 56 h, the molecular weights were equal to 44%^57^. Graphene/PCL composites were investigated by Murray et al.^58^ in a lipase solution at 37 °C for 4 days. Their results indicated a weight loss of 50%-60% within the first 24 h for thin films made from PCL alone and graphene/ poly(ε-caprolactone) composite films. The degradation kinetics of the compound in our study was lower than those from preceding studies^57,58^. It may be explained by the fact that the PCL scaffolds were subjected to a surface erosion mechanism, resulting in the loss of the amorphous zones on the scaffold's surface. The degradation of scaffolds and the formation of bone are closely linked processes since the destruction of scaffolds makes it easier for new bone tissue to grow^59^. However, the degradation rate for all scaffolds was very low because of PCL's high dimensional stability. As observed in this study, the pH value of composite scaffolds increased due to the release of alkaline ions from HA. Moreover, the pH value decreased due to the release of acidic by-products of polymers^40^. The = O group in PCL becomes positively charged ions at pH = 7.4 (PBS)^56^. As suggested by Li et al., the swelling behavior of the composite is due to the increased electrostatic repulsion between these ionized groups^60^. The degradation study revealed that, after 24 h of immersion in Lipase/PBS solution, an increase in the acidification of the media. One of the advantages of using HA in composite scaffolds is that they prevent swelling due to the degradation reaction of acidic polymers. The measured pH values for different materials indicate clear differences. As a result, the pH observed in composite scaffolds containing HA was about 7.2. By increasing the soaking time, the pH values of PCL/HA and PCL/HA/COL scaffolds soaked in PBS-Lipase increased at the early immersion stage. Also, the pH values of the composite scaffolds placed in PBS and PBS-Lipase increased after 14 days. However, the pure PCL scaffolds soaked in PBS and PBS-Lipase showed lower pH values compared to composite scaffolds.

We investigated the effect of post-fabricationmodification of 3D-printed PCL scaffolds with HA and COL on the cell proliferation and osteogenesis differentiation of hADSCs. Attachment of cells to the surface of scaffolds, growth, and proliferation was investigated several times after cell seeding by MTT and DAPI staining assays. As a result, the coating of 3D-printed PCL scaffolds with HA and COL enhances cell adhesion and proliferation. In a 2020 study, Mondal et al. demonstrated the effects HAp-modified PLA scaffolds and found that surfaces of the PLA/HAp scaffolds affected the response and proliferation of MG-63 cells^61^. MTT assay showed non-toxic properties of scaffolds materials. DNA molecules that contain A-T-rich areas are stained with DAPI^62^. Using fluorescence microscope, the nuclei of the cells can be observed after staining. These results are related to the coating of HA and COL on the scaffolds. DAPI staining displayed poor cell adhesion to PCL scaffolds, while composite scaffolds were covered with more cells, indicating better adhesion to composite scaffolds.

The bioactivity of a particular bone substitute for implantation and its capacity to stimulate the characteristic mending preparation inside the encompassing tissue can be upgraded by creating of HA evidence on the scaffold. It is because the osteoprogenitor cells, which are the shape of mesenchymal stem cells, and osteoblasts are more likely to attach to a mineralized surface, which is rougher than a smooth surface with the assistance of thrombospondin and vitronectin. Subsequently, through mineralized scaffolds, the quality of bone recovery and cell movement improved^63^. The mineralization of the scaffolds can enhance their mechanical properties and make it more possible to enhance stem cells and/or progenitor cells' differentiation into osteoblastic cells^63,64^. ALP is a key early differentiation marker for immature osteoblast activity that converts organic pyrophosphate to inorganic phosphate^65–68^. ALP, which is also involved in the formation of calcium apatite, is present in the bone matrix^66^. Our results showed that the ALP concentration of hADSCs cultured on scaffolds gradually increased from day 1 to 14. Compared with the PCL/COL and pure PCL scaffold, PCL/HA and PCL/HA/COL groups showed higher ALP activity. It seems that with the HA coated on composite scaffolds, the ALP activity of cells was gradually increased. Results show that both ALP expression and ALP activity increased over time. Osteonectin is strongly expressed at the beginning of bone differentiation, but its expression reduces with the acquisition of adult osteoblasts^69^. Osteonectin protein, also known as a calcium-binding glycoprotein, mediates the interaction between the differentiated osteoblasts and the matrix in normal skeletal tissue^66^. The expressions of osteonectin gene and osteonectin protein secretion for PCL/HA and PCL/HA/COL scaffolds are significantly higher than pure PCL scaffolds. The osteogenic behavior of composite scaffolds improved by using bioactive biomaterials, bioceramics, and natural polymers.

## Conclusion

In summary, 3D porous PCL scaffolds were fabricated using the FDM-3D printing method. The surface modification on the fabricated PCL scaffolds was successfully performed using HA and COL in the next step. The Physicochemical, molecular, and biological tests indicated that the PCL-based composites with modified HA and COL might be an ideal implant to stimulate hard tissue regeneration. The interaction of HA and COL caused enhanced cell attachment and proliferation. By combining 3D printing technology, unique design scaffolds, bioactive materials, cells, and growth factors, scaffolds can be tailored to the specific needs of patients and offer a new generation of bone tissue engineering scaffolding. One of the major challenges in bone tissue engineering is angiogenesis in newly developed tissue. However angiogenic stimuli in scaffolds,have remained an unsolved challenge.

## Materials and methods

### Materials

PCL with a molecular weight (Mw) of 80,000 g·mol^−1^, HA, dimethylsulfoxide (DMSO), 4',6-diamidino-2-phenylindole (DAPI), 3-(4,5-dimethylthiazol-2-yl)-2,5-diphenyl tetrazolium bromide (MTT) were purchased from Sigma-Aldrich, St. Louis MO. Trypsin/EDTA solution (0.25%), phosphate-buffered saline (PBS), fetal bovine serum (FBS), High glucose Dulbecco's minimum essential medium (DMEM), antibiotics (penicillin/streptomycin), 100 units/mL were obtained from Gibco, Burlington, ON, Canada. Acetic acid, sodium hydroxide (NaOH), ethanol, isopropanol, chloroform, dimethylformamide (DMF), glutaraldehyde, ARS, and Triton 100X were all bought from Merck, Darmstadt, Germany. An aqueous solution of collagen type-Ι (COL) that was prepared from porcine skin in acetic acid (CH3COOH) was supplied by Pasteur Institute of Iran. Lipase pseudomonas cepacia (Grade 62,309-100 mg) was procured from Sigma, Aldrich, St. Louis MO. An alkaline phosphatase (ALP) activity, total protein, and calcium content kits were also obtained from PARS-AZMUN, Tehran, IRAN.

### 3D PCL scaffold fabrications

The porous 3D scaffolds were manufactured by 3D printing using software (Repetier Host V2.1.3, 2011_2018).

PCL granules were filled into a heating cylinder, heated to a liquid phase, and removed through 1 mm slot nozzles by 3D melt extrusion/FDM with a 0°/90° lay-down pattern, forming square pores. The dimensions of the scaffold in z, y, and x coordinates were controlled using the machine when printing. The melted PCL was built layer by layer before the layers were joined. Printing parameters for making circular four-layer structures (diameter 15 mm), (thickness 0.5 mm) were adjusted as follows: a temperature of 110, 0.5 mm nozzle diameter, print speed at 2 mm per second, the distance between the two strands 0.3 mm and 0.2 mm layer thickness. 3D porous circle-shaped scaffolds were fabricated in two different sizes: 6(D) _12(H) mm for compression test and 15(D)_0.8(H) mm for in vitro studies.

### Surface modification

#### Postfabrication modification

Plasma treatment was used to improve the hydrophilicity of the surface of PCL scaffolds (a low-frequency plasma generator at 90 GHz, pure oxygen gas, 0.4 mbar pressure; ignition of glow discharge for 3 min, Plasma cleaner Zepto Model 1 base unit type A incl, Germany). Then, the 3D PCL scaffolds were treated for 10 min with 0.2 wt% NaOH solution and then washed three times with DI water to remove the remaining NaOH. The mesh scaffold method was used for HA and COL combination with PCL. The 3D PCL and PCL/HA scaffolds were immersed in a 2/5% glutaraldehyde solution in water for 20 min within a closed chamber to ensure that the membranes bonded under glutaraldehyde. The collagen becomes stronger and more stable through this process.

#### Hydroxyapatite and collagen coating

HA powders (1%, w/w) were suspended in 100 ml of distilled water for 30 min using an ultrasonic bath for 20 min at 37 °C to well dispersed during stirring. Plasma-treated scaffolds were immersed into the solution for HA coating and kept in the incubator overnight. For COL coating, the 3D PCL and PCL/HA scaffolds were immersed into a COL solution (10 mg/mL in 0.05 M acetic acid) for 24 h. In total, four groups of scaffolds were prepared after surface modification: PCL, PCL/COL, PCL/HA, and PCL/HA/COL.

### Scaffolds characterization

Attenuated Total Reflectance-Fourier Transform Infrared (ATR-FTIR) spectroscopy (Equinox 55, Bruker, Germany) was used to confirm the presence of HA and COL on the surface of PCL scaffolds. The chemical composition of scaffolds was also characterized using X-ray powder diffraction (XRD) (ARL X’TRA, Thermo Electron, USA). Diffraction data were collected in the range of 2θ from the accepted manuscript up to 80° using monochromatic CuKα radiation. Digital images of the fabricated scaffolds were captured (Nikon D3100, Nikon Corporation). Field emission scanning electron microscopy (FeSEM, Vega, Tescan, Czech Republic) equipped with Energy Dispersive X-ray (EDX) spectroscopy was used to characterize the surface morphology of the fabricated scaffolds. Moreover, Scaffold pore size was measured by Image J software (version 1.53a).

The compressive moduli of the fabricated scaffolds (diameter 6 mm and height 12 mm) were evaluated using a strength measuring device made by Santam KN 25 and according to ASTM F451-99a standard 100 N once at a speed of 1 mm/min, up to a maximum pressure of 80%. In order to determine the swelling properties of the fabricated scaffolds, the pre-weighed scaffolds were immersed in PBS at a temperature of 37° C for 1, 2, 4, 6, and 24 h. The water of every single scaffold was absorbed with a paper towel and dried in an oven, and weighed. Finally, the following equation was used to calculate the percentage of swelling:

$$Swelling\;ratio\left( \% \right) = \left[ {\frac{{W_{0} - W_{t} }}{{W_{0} }}} \right] \times 100$$

where W_0_ and W_t_ are the main weight and the wet weight of specimens, respectively.

Following characterization tests, a degradation test was also used to evaluate the weight loss of scaffolds. Samples were weighed in each group before destruction. The scaffolds were soaked at 37 °C with a rotational speed of 28 rpm (Thermoshaker, LS-100, Thermo Scientific, USA) for 28 days after incubation in PBS (0.01 M, pH = 7.4) under catalyzed conditions using lipase containing PBS. PBS and PBS-Lipase were changed every 3 days. The Lipase enzyme prepared from Pseudomonas cepacia was solved in PBS at a 0.5 mg/ml concentration. The weight of the scaffolds was measured before and after immersion in each degradation solution. The PCL scaffold was considered a control. The scaffolds were washed with ddH_2_O and dried in an oven for 24 h before the loss in mass of each scaffold was examined. The loss in mass of each scaffold was calculated as stated in the equation below:

$$Weight\, loss\left( \% \right) = \left[ {\frac{{W - W_{t} }}{W}} \right] \times 100$$

Finally, fabricated scaffolds were characterized using fluid movement to measure their porosities. Since ethanol can penetrate across the pores of the scaffold easily, it was used to make the moving fluid. Therefore, immersed the scaffold into a graduated container filled with ethanol (v_1_) until it floated. Volumes of total ethanol and saturated scaffolds were recorded as (v_2_). In the next step, the saturated scaffold was removed with ethanol. Then the volume of residual ethanol was recorded as (v_3_). The overall volume of the scaffold was calculated using the following equation:

$$v = \left( {v_{2} - v_{1} } \right) + \left( {v_{1} - v_{3} } \right) = v_{2} - v_{3}$$

In this equation, a polymeric scaffold's initial volume is (v_2_ − v_1_), and (v_1_ − v_3_) represents the volume of ethanol absorbed by the scaffold. Finally, the scaffold's porosity rate was measured using the following equation:

$$Porosity = \frac{{\left( {v_{1} - v_{3} } \right)}}{{\left( {v_{2} - v_{3} } \right)}}$$

### Cell culture and biocompatibility

For sterilization, the scaffolds were immersed in 70% alcohol for 1 h and then washed with PBS containing 1% antibiotic (penicillin–streptomycin) 3 times for 5 min. Finally, both sides of the scaffold were sterilized to ultraviolet radiation for 20 min. hADSCs prepared from the Iranian Biological Resource Center of Iran were used in this study. hADSCs were grown in DMEM (Dulbecco's Modified Eagle Medium) medium containing 10% FBS, 1% penicillin–streptomycin at 37° C, 5% CO_2_ and 95% humidity. The culture medium was changed every 3 days. Third passage cells were used for seeding on the sterilized scaffolds.

For the MTT assay, 1 × 10^4^ hADSCs were seeded in the fabricated scaffolds. After 1, 3, 7, 14 days of incubation, prepared seeded scaffolds were washed with PBS, and the culture medium was replaced with 200 μl DMEM containing 5 mg/ml 3-(4,5-dimethylthiazol-2-yl)-2,5-diphenyl tetrazolium bromide (MTT) and then incubated for 4 h at 37 °C. The medium was removed and replaced with Dimethyl sulfoxide (DMSO) (100 μl) to dissolve the purple formazan crystals. To better dissolve the MTT residue, the plate was placed on a shaker for 15 min, and then the dissolved precipitate was transferred to a 96-well cell culture dish. In this test, three wells containing cells and culture medium without samples were considered negative controls. Finally, samples were measured at 570 nm using the microplate reader (ELx 800, BioTek).

### Osteo-differentiation assays

To determine the osteosupportive capacity of the scaffolds, hAMSCs were cultured in an osteogenic medium containing DMEM supplemented with 10% FBS, dexamethasone, ascorbic acid, and β-glycerophosphate for 21 days. DMEM is most appropriate for supporting the growth of adherent cell phenotypes.

#### Alizarin red staining

Calcium deposition of the differentiated hADSCs on the fabricated scaffolds was assessed using Alizarin red staining (ARS) during the different periods (14 and 21 days). The seeded scaffolds were washed twice with PBS (1x) and then fixed for 15 min at room temperature using 4% paraformaldehyde. After that, fixed samples were rewashed by PBS and stained with a 2% alizarin red solution for 30 min. Then, all samples were washed with DI water and morphologically studied using the inverted light microscope (Olympus CKX41, Tokyo, Japan). The cells cultured in monolayer without scaffolds were used as a control.

#### Calcium content

Calcium extraction was performed to quantitatively measure the deposited calcium using 0.6 N HCL (Merck). After 1, 7, 14, and 21 days of incubation, hADSCs seeded scaffolds were washed with PBS and kept for 45 min in 500 μl, 0.6 N HCL solution. Then, according to the PARS-AZMUN calcium kit protocol (PARS-AZMUN), 20 μl of lysate was added to 1 ml of freshly prepared reagent and then transferred to a transparent 96-well plate. The calcium deposition of each group was measured at 570 nm using a microplate reader.

#### Alkaline phosphatase activity

After 1, 7, and 14 days of incubation, hADSCs seeded scaffolds were washed with PBS. The cells were then lysed in a cell lysis buffer, and the contents of each well were transferred to a separate microtube and kept on a shaker for 20 min at 4 °C. After that, the microtubes were centrifuged at 15,000 rpm for 15 min. The total protein supernatants were collected and then measured based on the total protein kit protocol (PARS-AZMUN, Iran). The optical density of ALP and total protein was measured at 405 nm with a microplate reader. ALP values were reported as ALP concentrations normalized with total protein content. One of the wells with the same amount of scaffold-free cells with a differentiation medium was considered a control.

#### Gene expression

*Osteonectin* and *ALP* bone genes were quantified in the hADSCs cultured on the fabricated scaffolds evaluated by quantitative real-time PCR (qRT-PCR). Total RNA was extracted with an RNA extraction kit (RNeasy Mini Kit, Qiagen, USA), and cDNA synthesis was performed using the Revert Aid first-strand cDNA synthesis kit on day 21. For real-time PCR, all reactions were performed under identical conditions, including 40 cycles of amplification with denaturation at 95 °C for 30 s, annealing at 60 °C for 20 s, and 30 s elongation at 72 °C for 40 cycles. Melting curve analysis and gel electrophoresis were used to determine the specificity of each primer set. The Ct values were normalized to the average Ct value of the housekeeping gene β-actin. The relative gene expression of composite scaffolds was analyzed by the log2-fold change (-ΔΔCt) method and compared with the expression in pure PCL scaffolds. Oligonucleotide primers for qRT-PCR amplifications are shown in Table 2.

**Table 2:** Oligonucleotide primers for quantitative real-time PCR amplifications.

Gene	Forward sequence	Revers sequence
*ALP*	GCCTTGCTCACTCACTCACT	ACAGGAGAGTCGCTTCAGAGA
*Osteonectin* (OSN)	ACATCGGGCCTTGCAAATAC	GTTGTCCTCATCCCTCTCAT
*Β-actin*	AGCACAGAGCCTCGCCTT	CACGATGGAGGGGAAGAC

#### Immunocytochemistry

hADSCs seeded scaffolds were analyzed after 21 days of incubation to express Osteonectin and ALP protein markers through ICC. The scaffolds were washed with PBS and fixed with paraformaldehyde %4 at 4 °C for 20 min. Then, the samples were washed twice with PBS. The immobilized cells were immersed in goat serum for 45 min. They were then immersed in Triton solution (4%) for 5 min. Primary Osteonectin antibodies (Abcam, UK) and ALP were incubated overnight at 4 °C and washed twice with PBS. Finally, 4′,6-diamidino-2-phenylindole (DAPI) (Sigma Chemical Company, USA) was added to label the cell nuclei and incubated for 30 s. They were washed twice with PBS. Immunofluorescence images were obtained using fluorescence microscopy (FV500, Olympus Fluoview, Japan), and prepared images were semi-quantified using Image J software.

### Statistical analysis

GraphPad, Prism software (V.9, USA) was used to perform one-way and two-way ANOVA for statistical comparisons of results. The probability values less than 0.05 (p-value < 0.05) were considered significant.

### Ethical approval

No human or animals used in this study.

## Data Availability

The datasets used and/or analyzed during the current study are available from the corresponding author on reasonable request.

## References

[1] Neumann, R., Neunzehn, J., Hinüber, C., Flath, T., Schulze, F. & Wiesmann, H. 3D-printed poly-ε-caprolactone-CaCO 3-biocompositescaffolds for hard tissue regeneration. *eXPRESS Polym Lett*. **13**, 2–7 (2019).

[2] Bose S, Sarkar N, Banerjee D (2018). Effects of PCL, PEG and PLGA polymers on curcumin release from calcium phosphate matrix for in vitro and in vivo bone regeneration. Mater. Today Chem..

[3] Alksne M (2020). In vitro comparison of 3D printed polylactic acid/hydroxyapatite and polylactic acid/bioglass composite scaffolds: Insights into materials for bone regeneration. J. Mech. Behav. Biomed. Mater..

[4] Battafarano G (2021). Strategies for bone regeneration: from graft to tissue engineering. Int. J. Mol. Sci..

[5] Huang B, Vyas C, Byun JJ, El-Newehy M, Huang Z, Bártolo P (2020). Aligned multi-walled carbon nanotubes with nanohydroxyapatite in a 3D printed polycaprolactone scaffold stimulates osteogenic differentiation. Mater. Sci. Eng. C..

[6] Klimek K, Ginalska G (2020). Proteins and peptides as important modifiers of the polymer scaffolds for tissue engineering applications—A review. Polymers.

[7] Grémare A (2018). Characterization of printed PLA scaffolds for bone tissue engineering. J. Biomed. Mater. Res. A..

[8] Witzler M (2019). Non-cytotoxic agarose/hydroxyapatite composite scaffolds for drug release. Int. J. Mol. Sci..

[9] Zhang W (2019). Fabrication and characterization of porous polycaprolactone scaffold via extrusion-based cryogenic 3D printing for tissue engineering. Mater. Des..

[10] Cestari F, Petretta M, Yang Y, Motta A, Grigolo B, Sglavo VM (2021). 3D printing of PCL/nano-hydroxyapatite scaffolds derived from biogenic sources for bone tissue engineering. Sustain. Mater. Technol..

[11] Guvendiren M, Molde J, Soares RM, Kohn J (2016). Designing biomaterials for 3D printing. ACS Biomater. Sci. Eng..

[12] Belaid H (2020). Boron nitride based nanobiocomposites: design by 3D printing for bone tissue engineering. ACS Appl. Biol. Mater..

[13] Edgar J, Tint S (2015). Additive manufacturing technologies: 3D printing, rapid prototyping, and direct digital manufacturing. Johnson Matthey Technol. Rev..

[14] Kafle A, Luis E, Silwal R, Pan HM, Shrestha PL, Bastola AK (2021). 3D/4D Printing of polymers: Fused deposition modelling (FDM), selective laser sintering (SLS), and stereolithography (SLA). Polymers.

[15] Siddiqui N, Asawa S, Birru B, Baadhe R, Rao S (2018). PCL-based composite scaffold matrices for tissue engineering applications. Mol. Biotechnol..

[16] Shapourzadeh A, Atyabi S-M, Irani S, Bakhshi H (2020). Osteoinductivity of polycaprolactone nanofibers grafted functionalized with carboxymethyl chitosan: Synergic effect of β-carotene and electromagnetic field. Int. J. Biol. Macromol..

[17] Rosales-Ibáñez R, Cubo-Mateo N, Rodríguez-Navarrete A, González-González AM, Villamar-Duque TE, Flores-Sánchez LO (2021). Assessment of a PCL-3D printing-dental pulp stem cells triplet for bone engineering: An in vitro study. Polymers.

[18] Karimzadeh Bardeei L, Seyedjafari E, Hossein G, Nabiuni M, Majles Ara MH, Salber J (2021). Regeneration of bone defects in a rabbit femoral osteonecrosis model using 3D-printed poly (epsilon-caprolactone)/nanoparticulate willemite composite scaffolds. Int. J. Mol. Sci..

[19] Jiao Z (2019). 3D printing of HA/PCL composite tissue engineering scaffolds. Adv. Ind. Eng. Polym. Res..

[20] Hassan MI, Sultana N (2017). Characterization, drug loading and antibacterial activity of nanohydroxyapatite/polycaprolactone (nHA/PCL) electrospun membrane. 3 Biotech..

[21] Kim CG, Han KS, Lee S, Kim MC, Kim SY, Nah J (2021). Fabrication of biocompatible polycaprolactone-hydroxyapatite composite filaments for the FDM 3D printing of bone scaffolds. Appl. Sci..

[22] Hashemi S (2021). Comparison of osteogenic differentiation potential of induced pluripotent stem cells and buccal fat pad stem cells on 3D-printed HA/β-TCP collagen-coated scaffolds. Cell Tissue Res..

[23] Rajzer I, Menaszek E, Kwiatkowski R, Planell JA, Castano O (2014). Electrospun gelatin/poly (ε-caprolactone) fibrous scaffold modified with calcium phosphate for bone tissue engineering. Mater. Sci. Eng. C.

[24] He, Y., *et al.* A 3D-printed PLCL scaffold coated with collagen type I and its biocompatibility. *BioMed. Res. Int*. **2018**, (2018). 10.1155/2018/5147156 PMC5911326 29850530

[25] Li Q (2019). Hydroxyapatite/collagen three-dimensional printed scaffolds and their osteogenic effects on human bone marrow-derived mesenchymal stem cells. Tissue Eng. Part A.

[26] Patil VA, Masters KS (2020). Engineered collagen matrices. Bioengineering.

[27] Li H, Tan C, Li L (2018). Review of 3D printable hydrogels and constructs. Mater. Des..

[28] Leach JK, Whitehead J (2017). Materials-directed differentiation of mesenchymal stem cells for tissue engineering and regeneration. ACS Biomater. Sci. Eng..

[29] Lynch CR, Kondiah PP, Choonara YE (2021). Advanced strategies for tissue engineering in regenerative medicine: A biofabrication and biopolymer perspective. Molecules.

[30] Yu J (2020). Fabrication of a polycaprolactone/alginate bipartite hybrid scaffold for osteochondral tissue using a three-dimensional bioprinting system. Polymers.

[31] Mystiridou E, Patsidis AC, Bouropoulos N (2021). Development and characterization of 3D printed multifunctional bioscaffolds based on PLA/PCL/HAp/BaTiO3 composites. Appl. Sci..

[32] Goncalves EM (2016). Three-dimensional printed PCL-hydroxyapatite scaffolds filled with CNT s for bone cell growth stimulation. J. Biomed. Mater. Res. Part B Appl. Biomater..

[33] Kim MH (2018). Quantitative analysis of the role of nanohydroxyapatite (nHA) on 3D-printed PCL/nHA composite scaffolds. Mater. Lett..

[34] Pavon C, Aldas M, López-Martínez J, Ferrándiz S (2020). New materials for 3D-printing based on polycaprolactone with gum rosin and beeswax as additives. Polymers.

[35] Aidun A (2019). Graphene oxide incorporated polycaprolactone/chitosan/collagen electrospun scaffold: Enhanced osteogenic properties for bone tissue engineering. Artif. Organs..

[36] Goodarzi H, Hashemi-Najafabadi S, Baheiraei N, Bagheri F (2019). Preparation and characterization of nanocomposite scaffolds (collagen/β-TCP/SrO) for bone tissue engineering. J. Tissue Eng. Regen. Med..

[37] Holländer J (2016). Three-dimensional printed PCL-based implantable prototypes of medical devices for controlled drug delivery. J. Pharm. Sci..

[38] Kim Y (2019). 3D-printed PCL/bioglass (BGS-7) composite scaffolds with high toughness and cell-responses for bone tissue regeneration. J. Ind. Eng. Chem..

[39] Perez-Puyana V (2021). Fabrication of hybrid scaffolds obtained from combinations of PCL with gelatin or collagen via electrospinning for skeletal muscle tissue engineering. J. Biomed. Mater. Res. Part A..

[40] Hassanajili S, Karami-Pour A, Oryan A, Talaei-Khozani T (2019). Preparation and characterization of PLA/PCL/HA composite scaffolds using indirect 3D printing for bone tissue engineering. Mater. Sci. Eng. C.

[41] Wang C (2020). 3D printing of bone tissue engineering scaffolds. Bioact. Mater..

[42] Weems AC, Pérez-Madrigal MM, Arno MC, Dove AP (2020). 3D printing for the clinic: Examining contemporary polymeric biomaterials and their clinical utility. Biomacromol.

[43] Yu J, Xu Y, Li S, Seifert GV, Becker ML (2017). Three-dimensional printing of nano hydroxyapatite/poly (ester urea) composite scaffolds with enhanced bioactivity. Biomacromol.

[44] Babilotte J, Guduric V, Le Nihouannen D, Naveau A, Fricain JC, Catros S (2019). 3D printed polymer–mineral composite biomaterials for bone tissue engineering: fabrication and characterization. J. Biomed. Mater. Res. Part B Appl. Biomater..

[45] Rashad A (2018). Coating 3D printed polycaprolactone scaffolds with nanocellulose promotes growth and differentiation of mesenchymal stem cells. Biomacromol.

[46] Karimi Z (2019). Baghdadite nanoparticle-coated poly l-lactic acid (PLLA) ceramics scaffold improved osteogenic differentiation of adipose tissue-derived mesenchymal stem cells. J. Biomed. Mater. Res. Part A.

[47] Mashhadikhan M, Soleimani M, Parivar K, Yaghmaei P (2015). ADSCs on PLLA/PCL hybrid nanoscaffold and gelatin modification: Cytocompatibility and mechanical properties. Avicenna J. Med. Biotechnol..

[48] Park S (2021). 3D-printed poly (ε-caprolactone)/hydroxyapatite scaffolds modified with alkaline hydrolysis enhance osteogenesis in vitro. Polymers.

[49] Turnbull G (2018). 3D bioactive composite scaffolds for bone tissue engineering. Bioact. Mater..

[50] Zhang D, Wu X, Chen J, Lin K (2018). The development of collagen based composite scaffolds for bone regeneration. Bioact. Mater..

[51] Lee C-F (2021). 3D printing of collagen/oligomeric proanthocyanidin/oxidized hyaluronic acid composite scaffolds for articular cartilage repair. Polymers.

[52] Jayatissa NU, Unagolla J, Bhaduri S (2018). 3D printed polymer scaffolds for bone tissue engineering. Ohio J. Sci..

[53] Marouf N, Nojehdehian H, Ghorbani F (2020). Physicochemical properties of chitosan–hydroxyapatite matrix incorporated with Ginkgo biloba-loaded PLGA microspheres for tissue engineering applications. Polym. Polym. Compos..

[54] Moghadam MZ, Hassanajili S, Esmaeilzadeh F, Ayatollahi M, Ahmadi M (2017). Formation of porous HPCL/LPCL/HA scaffolds with supercritical CO2 gas foaming method. J. Mech. Behav. Biomed. Mater..

[55] Ligon SC, Liska R, Stampfl J, Gurr M, Mülhaupt R (2017). Polymers for 3D printing and customized additive manufacturing. Chem. Rev..

[56] Trakoolwannachai V, Kheolamai P, Ummartyotin S (2019). Characterization of hydroxyapatite from eggshell waste and polycaprolactone (PCL) composite for scaffold material. Compos. B Eng..

[57] Brugmans M (2015). Hydrolytic and oxidative degradation of electrospun supramolecular biomaterials: In vitro degradation pathways. Acta Biomater..

[58] Murray E, Thompson BC, Sayyar S, Wallace GG (2015). Enzymatic degradation of graphene/polycaprolactone materials for tissue engineering. Polym. Degrad. Stab..

[59] Raina DB (2018). Gelatin-hydroxyapatite-calcium sulphate based biomaterial for long term sustained delivery of bone morphogenic protein-2 and zoledronic acid for increased bone formation: In-vitro and in-vivo carrier properties. J. Controlled Release..

[60] Li B (2016). Protein-cross-linked hydrogels with tailored swelling and bioactivity performance: A comparative study. ACS Appl. Mater. Interfaces.

[61] Mondal S (2020). Hydroxyapatite nano bioceramics optimized 3D printed poly lactic acid scaffold for bone tissue engineering application. Ceram. Int..

[62] Lee C, Kim Y-J, Kim KS, Lee JY, Kim D-N (2021). Modulating the chemo-mechanical response of structured DNA assemblies through binding molecules. Nucleic Acids Res..

[63] Wu X, Walsh K, Hoff BL, Camci-Unal G (2020). Mineralization of biomaterials for bone tissue engineering. Bioengineering.

[64] Zou L, Zhang Y, Liu X, Chen J, Zhang Q (2019). Biomimetic mineralization on natural and synthetic polymers to prepare hybrid scaffolds for bone tissue engineering. Colloids Surf. B.

[65] Arab-Ahmadi S, Irani S, Bakhshi H, Atyabi F, Ghalandari B (2021). Immobilization of carboxymethyl chitosan/laponite on polycaprolactone nanofibers as osteoinductive bone scaffolds. Polym. Adv. Technol..

[66] Orafa Z, Irani S, Zamanian A, Bakhshi H, Nikukar H, Ghalandari B (2021). Coating of laponite on PLA nanofibrous for bone tissue engineering application. Macromol. Res..

[67] Song, Y., Mou, R., Li, Y. & Yang T. Zingerone promotes osteoblast differentiation via MiR-200c-3p/smad7 regulatory axis in human bone mesenchymal stem cells. *Med. Sci. Monit*. **26**, e919309–1–e919309–10 (2020). 10.12659/MSM.919309 PMC7079314 32146478

[68] Wang Q, Ma Z, Wang Y, Zhong L, Xie W (2021). Fabrication and characterization of 3D printed biocomposite scaffolds based on PCL and zirconia nanoparticles. Bio-Des. Manuf..

[69] Ranganathan S, Balagangadharan K, Selvamurugan N (2019). Chitosan and gelatin-based electrospun fibers for bone tissue engineering. Int. J. Biol. Macromol..

